# Prediction model for postoperative delirium risk in elderly hypertensive patients: machine learning-based development and validation

**DOI:** 10.3389/fpsyt.2026.1851947

**Published:** 2026-06-03

**Authors:** Kun Wang, Zhengzheng Zhao, Jiayi Chen, Wenjie Kong, Yuanlong Wang, Yizhi Liang, Yanan Lin, Chuan Li, Jiahan Wang, Hongyan Gong, Bin Wang, Xu Lin, Yongxin Liang, Yanlin Bi

**Affiliations:** 1 Department of Anesthesiology, Shandong Second Medical University, Weifang, China; 2 Department of Pain Management, Qingdao Municipal Hospital, Qingdao, China; 3 The Second School of Clinical Medicine of Binzhou Medical University, Yantai, China; 4 Department of Anesthesiology, Qingdao Municipal Hospital, Qingdao, Shandong, China; 5 Peking University People’s Hospital, Women and Children’s Hospital, Qingdao University, Qingdao, Shandong, China

**Keywords:** aged, hypertension, machine learning, mortality, postoperative delirium

## Abstract

**Background:**

Postoperative delirium (POD) is a severe complication in elderly hypertensive patients, associated with poor long-term outcomes. Existing models often rely on intraoperative data, limiting preoperative risk stratification. This study aimed to develop a non-invasive machine learning model to predict POD and investigate its preoperative markers’ impact on three-year mortality.

**Methods:**

Preoperative variables were selected using LASSO regression from a cohort of 1,782 patients. Ten machine learning models were trained and validated (7:3 ratio). Model performance was evaluated via AUC-ROC and decision curve analysis (DCA). The optimal model was interpreted using SHAP values. Long-term prognosis within the POD cohort was assessed using Kaplan-Meier curves and multivariable Cox proportional hazards regression.

**Results:**

The POD incidence was 10.9%. The Gradient Boosting Machine (GBM) demonstrated optimal performance (AUC = 0.868, 95% CI: 0.819–0.917). SHAP analysis identified MMSE score as the most influential predictor, followed by HADS score, age, CFS, frailty, and PSQI score. Multivariable Cox analysis revealed that lower MMSE, alongside elevated HADS, CFS, frailty, and PSQI scores—but not chronological age—were independent predictors of increased three-year mortality in POD patients (all *P* < 0.05).

**Conclusion:**

We developed a robust machine learning tool for individualized POD prediction. Cognitive impairment, psychological distress, frailty, and poor sleep quality serve as critical dual-prognostic markers for both acute POD occurrence and long-term survival. These findings underscore the necessity of routine multidimensional preoperative assessment to facilitate personalized interventions for vulnerable hypertensive populations.

## Background

Postoperative delirium (POD) is one of the most common complications in surgical patients, characterized by acute impairment of attention, thinking, and cognitive function, with symptoms exhibiting diurnal fluctuations (1, 2). Among patients undergoing elective major surgery, the incidence of POD is approximately 15%–25%, while it can reach as high as 50% in high-risk surgeries (3, 4). POD is not only closely associated with prolonged hospital stays, poor functional recovery, and cognitive decline, but also significantly increases healthcare costs, readmission rates, and even postoperative mortality, imposing a heavy burden on patients, families, and the healthcare system (5–7). Therefore, accurately predicting and identifying high-risk patients for POD remains a significant challenge in current clinical practice.

The occurrence of POD is associated with multiple factors, including advanced age, hypertension, diabetes, and preoperative cognitive impairment (8). However, complex interactions exist among these factors, and there is currently a lack of simple and effective predictive tools for identifying high-risk patients (9). Clinically, multiple scoring scales (such as MMSE, PSQI, etc.) are commonly used to assess patients’ preoperative status and perform POD risk stratification (10, 11). However, these approaches still have limitations when addressing multidimensional, nonlinear clinical characteristics, such as polypharmacy, frailty, and sensory impairments, in elderly patients (12).

In recent years, machine learning methods based on multidimensional data analysis have demonstrated significant potential in disease prediction by effectively integrating complex variable interactions (13, 14). These advanced approaches overcome the limitations of traditional models in capturing nonlinear relationships, thereby providing new directions for constructing highly accurate POD prediction tools.

Hypertension, as one of the most common chronic diseases, has a clear pathophysiological link to cognitive impairment (15). Elderly hypertensive patients often exhibit impaired cerebral vasomotor regulation and neurovascular unit dysfunction, which may reduce their tolerance to surgical and anesthetic stress, thereby increasing the risk of postoperative hypotension (16, 17). Although research on POD prediction models has made some progress, current models for predicting POD in elderly hypertensive patients remain under-explored (18). General prediction models may not accurately reflect the risk characteristics of postoperative delirium in this population.

To this end, this study aims to identify independent risk factors for POD in elderly hypertensive patients and to develop and validate a POD risk prediction model tailored to this population using machine learning algorithms. By systematically screening key predictive variables and evaluating their predictive value for POD and three-year postoperative mortality, we seek to provide a basis for the early and precise identification of high-risk patients and the formulation of personalized intervention strategies.

## Method

### Study population

This retrospective cohort study initially evaluated a consecutive series of patients who underwent elective non-cardiac surgery under general anesthesia with endotracheal intubation at Qingdao Municipal Hospital between January 2020 and June 2022, totaling 2,000 candidates. The study protocol was approved by the Institutional Ethics Committee, and the requirement for informed consent was waived due to the retrospective and anonymous nature of the data analysis. Eligibility criteria included patients with a documented diagnosis of hypertension, aged 65 years or older, with preserved preoperative cognitive status and sufficient educational background to independently complete cognitive and psychological assessments. Exclusion criteria were predefined as: (1) emergency surgery or major cardiovascular procedures performed within the preceding month; (2) a preoperative Mini-Mental State Examination (MMSE) score < 23 (this threshold was implemented to ensure patients possessed the cognitive capacity required to reliably respond to subjective questionnaires, such as HADS and PSQI, and to prevent the confounding of acute POD with baseline severe dementia); (3) history of significant neurological disorders, including central nervous system infections, traumatic brain injury, epilepsy, or multiple sclerosis; (4) uncontrolled cardiovascular or cerebrovascular conditions, or severe hepatic and renal insufficiency; (5) pre-existing psychiatric disorders or impaired consciousness; and (6) a history of substance abuse or chronic use of psychotropic or systemic corticosteroid medications.

### Ethical statement

This study adheres to the principles of the Declaration of Helsinki. The research protocol has been approved by the Ethics Committee of Qingdao Municipal Hospital (Approval No.: 2024-KY-094) and formally registered with the China Clinical Trial Registry (Registration No.: ChiCTR2500099473). All patient identities are replaced with hospital identification numbers and anonymized for confidentiality. Therefore, the Ethics Committee has granted exemption from written informed consent.

### Clinical baseline data collection

Qualified anesthesiologists collected clinical variables within 24 hours before surgery. Information gathered included patient gender, age, years of education, smoking status, alcohol consumption history, physical activity levels, and past medical history of hypertension, diabetes, and coronary heart disease. Smoking status was defined as current smoking (at least one cigarette daily for over one year); alcohol consumption history was defined as drinking at least once weekly for over one year. Physical activity was assessed by frequency and duration, with regular exercise defined as at least three sessions per week of moderate-intensity physical activity lasting 30 minutes or longer per session; a history of hypertension was defined as blood pressure ≥140/90 mmHg; Diabetes history was defined as meeting any of the following criteria: fasting venous blood glucose ≥7.0 mmol/L, random venous blood glucose ≥11.1 mmol/L, or 2-hour post-oral glucose tolerance test blood glucose ≥11.1 mmol/L. Coronary heart disease history was defined as coronary artery disease confirmed by coronary angiography or clinical manifestations (e.g., typical angina symptoms, history of myocardial infarction).

### Questionnaires and assessment tools

This study employed a series of validated standardized questionnaires to collect multidimensional health data. Sleep quality was assessed using the Pittsburgh Sleep Quality Index (PSQI; total score 0–21, >5 indicating poor sleep quality) and the Athens Insomnia Scale (AIS; total score 0–24, ≥6 suggesting insomnia), while subjective perceptions of sleep disturbance were quantified using a Numerical Rating Scale (NRS). Cognitive function was assessed using the Mini-Mental State Examination (MMSE; total score, 0–30, typically with a cutoff of <24 for cognitive impairment), and subjective complaints were evaluated using the Subjective Cognitive Decline (SCD) questionnaire. Nutritional status was assessed using the Mini Nutritional Assessment (MNA), categorized as: ≥24 points (nutritionally adequate), 17–23.5 points (at risk of malnutrition), and <17 points (malnourished). Psychological distress symptoms were screened using the Hospital Anxiety and Depression Scale (HADS), with subscale scores ≥8 indicating potential anxiety or depression. Frailty status was assessed using two tools: the Clinical Frailty Scale (CFS), which categorizes frailty severity into 1–9 levels (Level 1 being very healthy, Level 9 being terminal), and the Frail Scale, which performed rapid screening across five dimensions: fatigue, endurance, walking ability, illness, and weight loss. Physical activity levels were assessed using the International Physical Activity Questionnaire (IPAQ), with results expressed in MET-minutes per week.

### Primary outcome

The Confusion Assessment Method (CAM) was used to identify POD. Individuals testing positive for delirium were assigned to the POD group, while those testing negative were assigned to the non-POD (NPOD) group. All subjects were assessed by trained medical personnel unaware of their perioperative status between 1 and 7 days postoperatively.

### Follow-up

Survival time was measured from the date of surgery to either the occurrence of death or the most recent follow-up appointment. Investigators followed patients who developed POD for three years postoperatively.

### Data preprocessing

To ensure the integrity of the predictive model, clinical records with missing values were managed using a complete-case analysis approach. Prior to feature selection and model development, all continuous variables were standardized using Z-score normalization to attain a mean of zero and unit variance, ensuring that variables with different scales contributed proportionately to the models.

### Feature selection

Feature selection was performed using the Least Absolute Shrinkage and Selection Operator (LASSO) regression, implemented via the “glmnet” package in R. This method is particularly effective for high-dimensional clinical data as it penalizes regression coefficients, effectively shrinking non-essential predictors to zero and reducing the risk of overfitting.

To ensure the robustness of feature selection and prevent data leakage, the LASSO procedure was conducted exclusively within the training set. We employed a 10-fold cross-validation framework to determine the optimal regularization parameter (λ). The λ value that minimized the cross-validated deviance (lambda.min) was selected as the threshold for feature inclusion. Following this rigorous selection process, 14 predictors with non-zero coefficients were identified and subsequently utilized as the finalized input features for the development of all ten machine learning models.

### Machine learning models for POD prediction

The finalized dataset, comprising 1,782 patients, was randomly partitioned into a training set (70%, n = 1,247) for model construction and an independent testing set (30%, n = 535) for performance evaluation. To maintain consistency across the feature space, all machine learning algorithms were developed and validated utilizing only the 14 optimal predictors previously identified through the LASSO regression analysis.

Ten distinct machine learning algorithms were implemented and compared: a Neural Network (NN), Logistic Regression (LR), Support Vector Machine (SVM), Gradient Boosting Machine (GBM), Random Forest (RF), Extreme Gradient Boosting (XGBoost), K-Nearest Neighbours (KNN), AdaBoost, LightGBM, and CatBoost. To optimize model performance and mitigate overfitting, hyperparameter tuning was conducted exclusively within the training set using a grid-search strategy combined with 5-fold cross-validation. The predictive performance of each model was rigorously quantified on the testing set using the Area Under the Receiver Operating Characteristic Curve (AUC), accuracy, sensitivity, specificity, precision, and F1-score. Furthermore, the Brier score was calculated to assess model calibration, supplemented by visual inspection of calibration curves to evaluate the agreement between predicted probabilities and observed outcomes. The clinical utility and net benefit of the models were determined through Decision Curve Analysis (DCA), with confidence intervals and stability ensured via a bootstrap resampling method incorporating 50 iterations.

### SHAP analysis for model interpretation

To enhance the interpretability of the optimal machine learning model, this study employed the Shapley Additive exPlanations (SHAP) framework. This method quantifies the contribution of each feature to individual predictions by calculating its SHAP value, thereby elucidating both the magnitude and direction (positive or negative influence) of its effect on the prediction.

The analysis involved calculating SHAP values for each sample using the final predictive model. Global feature importance was then determined by aggregating the absolute SHAP values across all samples to rank the predictors. To visually communicate the complex interactions between risk factors and model outputs, a suite of graphical tools was employed, including summary plots for feature importance, dependence plots to illustrate feature-prediction relationships, and force plots to explain individual predictions.

### Development of a logistic regression model and clinical tools based on SHAP-identified core variables

Leveraging the core predictive variables identified through the SHAP analysis, we constructed a multivariate logistic regression model to estimate the probability of POD occurrence as a binary outcome.

To facilitate clinical translation, we developed a practical score-based nomogram derived from the model, which enables a visual assessment of POD risk. Furthermore, the model’s coefficients were implemented into an interactive web-based application, designed to provide clinicians with a tool for real-time, individualized risk calculation.

### Statistical analysis

The normality of continuous variables was assessed via the Kolmogorov-Smirnov test. Data conforming to a Gaussian distribution are expressed as mean ± standard deviation, whereas non-normally distributed data are presented as median (interquartile range). Intergroup comparisons of continuous variables were performed using independent samples t-tests for parametric data or Mann-Whitney U tests for non-parametric data. Categorical variables were compared employing χ² tests or Fisher’s exact tests as appropriate.

For the longitudinal prognosis analysis, Kaplan-Meier survival curves and Log-rank tests were initially employed to compare three-year mortality rates among different subgroups (e.g., Age, MMSE, HADS, CFS, Frail, and PSQI) within the POD cohort. To identify independent predictors of mortality while adjusting for potential confounders, a multivariable Cox proportional hazards regression analysis was performed. Variables demonstrated to be clinically significant or showing a *p*-value < 0.1 in univariate analyses were included in the multivariable model to calculate adjusted Hazard Ratios (HRs) and their corresponding 95% confidence intervals (CIs).

Statistical analyses were performed utilizing SPSS (version 25.0; IBM SPSS, Chicago, USA) and R (release 4.3.1; R Statistical Computing Foundation, Vienna, Austria). A significance threshold of *P* < 0.05 was applied for all tests. A data analysis and statistical plan was written after the data were accessed.

## Results

### Patient screening and grouping

**Figure 1:**
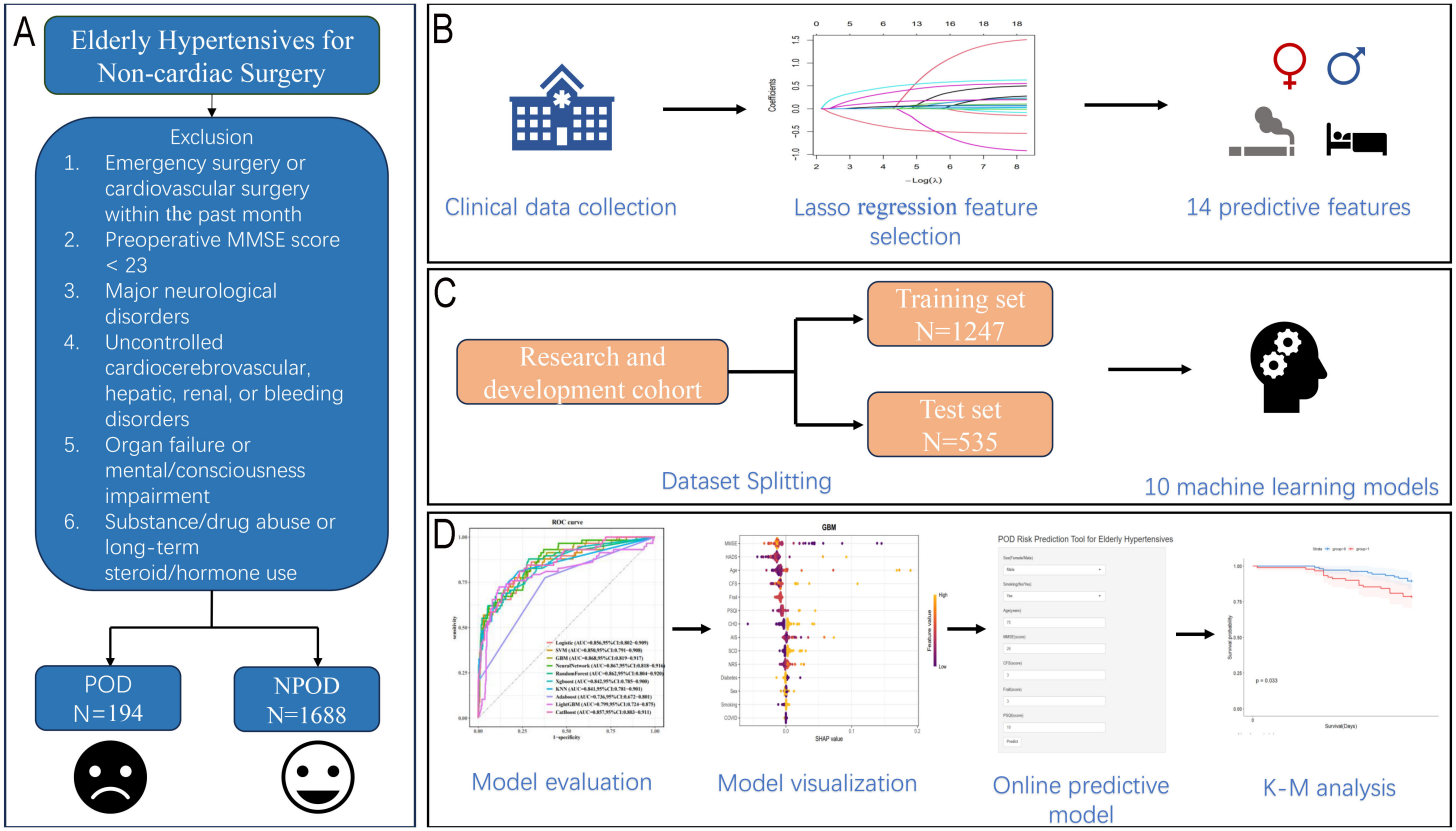
Patient screening process and study design diagram. **(A)** Patient screening process. **(B)** Data collection and feature selection flowchart. **(C)** The study cohort was divided into training and validation sets in a 7:3 ratio, followed by the development of ten machine learning models. **(D)** After model comparison, the best model was selected, visualized, and the risk factors contributing most to the outcome were identified. Based on these risk factors, survival analysis and nomogram model construction were further conducted.

A total of 2,000 patients were initially screened, of whom 218 (116 ineligible and 102 with missing clinical data) were excluded based on predefined criteria, resulting in 1,782 patients included in the final retrospective analysis (**Figure 1**; **Supplementary Figure 1**).

### Baseline clinical characteristics

This study included 1,782 elderly hypertensive patients, among whom 194 (10.9%) developed postoperative delirium (POD). These patients were compared with 1,588 patients in the non-POD group (see **Table 1**). Patients with POD were older and had higher NRS, HADS, PSQI, frailty, and CFS scores, as well as a higher prevalence of subjective cognitive decline, diabetes, coronary heart disease, and malnutrition. They also had fewer years of education, lower MMSE scores, and a lower proportion of physical activity (all *P* < 0.05). However, no significant differences were observed between the groups in terms of AIS score, MET score, gender, smoking, alcohol consumption, or history of COVID-19 infection (all *P* > 0.05).

**Table 1:** Demographic and characteristics of participants.

Variables	NPOD	POD	*P* value
	1588	194	
Age, M (IQR), years	70 (67-74)	74 (71-81)	**<0.001**
NRS, M (IQR), score	2 (1-3.75)	3 (2-4)	**0.030**
AIS, M (IQR), score	3 (2-4)	3 (1-5)	0.085
MET, M (IQR), score	3.5 (3-4)	3 (2-4)	0.540
Education, M (IQR), years	9 (6-10)	6 (5-9)	**<0.001**
MMES, M (IQR), score	27 (26-28)	25 (24-26)	**<0.001**
HADS, M (IQR), score	5 (3-6)	6.5 (4-13.0)	**<0.001**
PSQI, M (IQR), score	6 (4-9)	9 (6-12)	**<0.001**
Frail, M (IQR), score	1 (0-2)	2 (1-3)	**<0.001**
CFS, M (IQR), score	2 (1-3)	4 (3-5)	**<0.001**
Sex (female/male)	850/738	106/88	0.769
SCD, yes (%)	718 (45.2)	123 (63.4)	**<0.001**
Diabetes, yes (%)	529 (33.3)	91 (46.9)	**<0.001**
CHD, yes (%)	846 (53.3)	126 (64.9)	**0.002**
Cigarette smoking, yes (%)	324 (20.7)	35 (18.0)	0.439
Drinking, yes (%)	271 (17.1)	37 (19.1)	0.485
Physical activity, yes (%)	499 (31.4)	43 (22.2)	**0.008**
COVID, yes (%)	1515 (95.4)	187 (96.4)	0.530
MNA Category, n (%)			**<0.001**
Well-nourished	749 (47.2)	65 (33.5)	
At risk of malnutrition	651 (41.0)	65 (33.5)	
Malnourished	188 (11.8)	64 (33.0)	

POD, Postoperative delirium; NPOD, No postoperative delirium; PSQI, Pittsburgh Sleep Quality Index; AIS, Athens Insomnia Scaletotal; NRS, Numerical Rating Scale; MMSE, Mini-Mental State Examination; SCD, Subjective Cognitive Decline. MNA, Mini Nutritional Assessment; HADS, Hospital Anxiety and Depression Scale; CFS, Clinical Frailty Scale; CHD, Coronary heart disease; The bold values are all p ≤ 0.05, statistically significant parameters.

### Model construction and variable selection

The total cohort of 1,782 elderly hypertensive patients was randomly partitioned into a training set (n = 1,247) and a testing set (n = 535) at a 7:3 ratio. Comparative analysis revealed no statistically significant differences in baseline demographic or clinical characteristics between the two cohorts (**Table 2**), ensuring the internal validity of the subsequent modeling process.

**Table 2:** Comparison of demographic characteristics between the training dataset and the testing dataset.

Variables	Training set	Testing set	*P* value
	1247	535	
Age, M (IQR), years	70 (67-75)	71 (66-75)	0.202
NRS, M (IQR), score	2 (1-4)	2 (2-4)	0.435
AIS, M (IQR), score	3 (2-5)	3 (2-4)	0.164
MET, M (IQR), score	3 (3-4)	3 (3-4)	0.501
Education, M (IQR), years	9 (5-10)	9 (6-10)	0.830
MMES, score, M (IQR)	27 (25-28)	27 (25-28)	0.064
HADS, M (IQR), score	5 (3-6)	5 (3-6)	0.784
PSQI, M (IQR), score	6 (4-9)	6 (4-9)	0.267
Frail, M (IQR), score	1 (0-2)	1 (0-2)	0.365
CSF, M (IQR), score	3 (1-3)	2 (1-3)	0.929
POD, yes (%)	136 (10.9)	58 (10.9)	0.982
Sex (female/male)	568/680	258/276	0.277
SCD, yes (%)	609 (48.8)	232 (43.4)	**0.038**
Diabetes, yes (%)	435 (34.9)	185 (34.6)	0.932
CHD, yes (%)	681 (54.6)	291 (54.5)	0.977
Cigarette smoking, yes (%)	267 (21.4)	92 (17.2)	**0.045**
Drinking, yes (%)	221 (17.7)	87 (16.3)	0.469
Physical activity, yes (%)	373 (29.9)	169 (31.6)	0.459
COVID, yes (%)	1202 (96.3)	500 (93.6)	**0.012**
MNA Category, n (%)			0.715
Well-nourished	567 (45.4)	247 (46.3)	
At risk of malnutrition	499 (40.0)	217 (40.6)	
Malnourished	182 (14.6)	70 (13.1)	

The bold values are all *p* ≤ 0.05, statistically significant parameters.

For feature selection, we utilized LASSO regression analysis within the training set to mitigate potential overfitting and identify the most robust predictors for POD. The optimal regularization parameter (λ) was determined through 10-fold cross-validation, with the λ value (the threshold that minimizes cross-validated binomial deviance) selected to ensure peak predictive performance.

**Figure 2:**
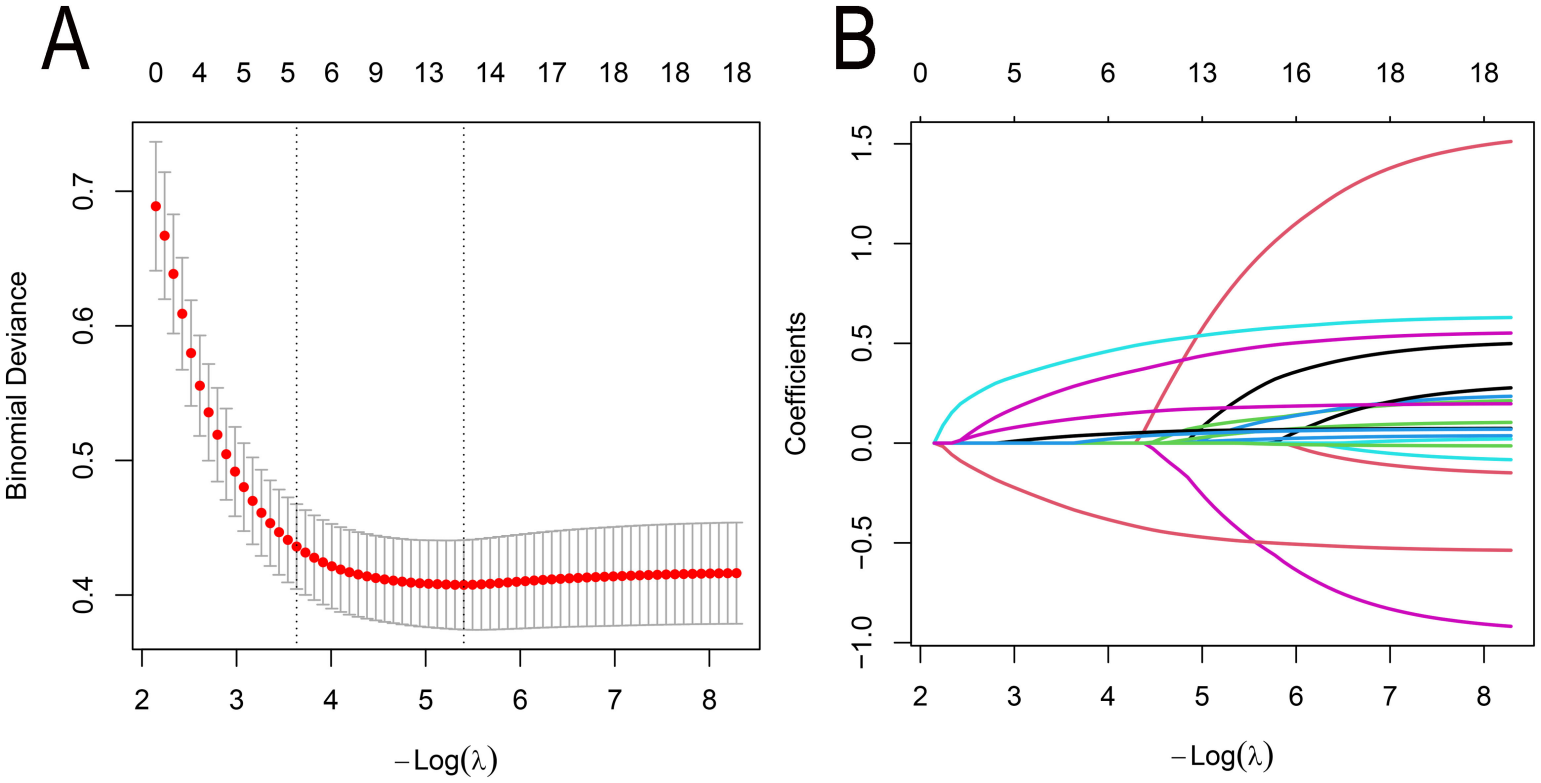
LASSO feature selection for model construction. **(A)** Binomial deviance as a function of the logarithm of the λ parameter for LASSO regression. **(B)** Coefficient path of the LASSO regression model as a function of the regularization parameter, λ. LASSO, Least Absolute Shrinkage and Selection Operator.

Following this procedure, 14 preoperative predictors with non-zero coefficients were identified from the initial variable pool: sex, smoking history, history of COVID-19 infection, age, CHD, diabetes, SCD, NRS, AIS, HADS, MMSE, PSQI, frailty, and CFS score (**Figure 2**). These 14 variables were consistently utilized as the finalized input feature set for the development and validation of all ten machine learning models.

### Model performance comparison

**Figure 3:**
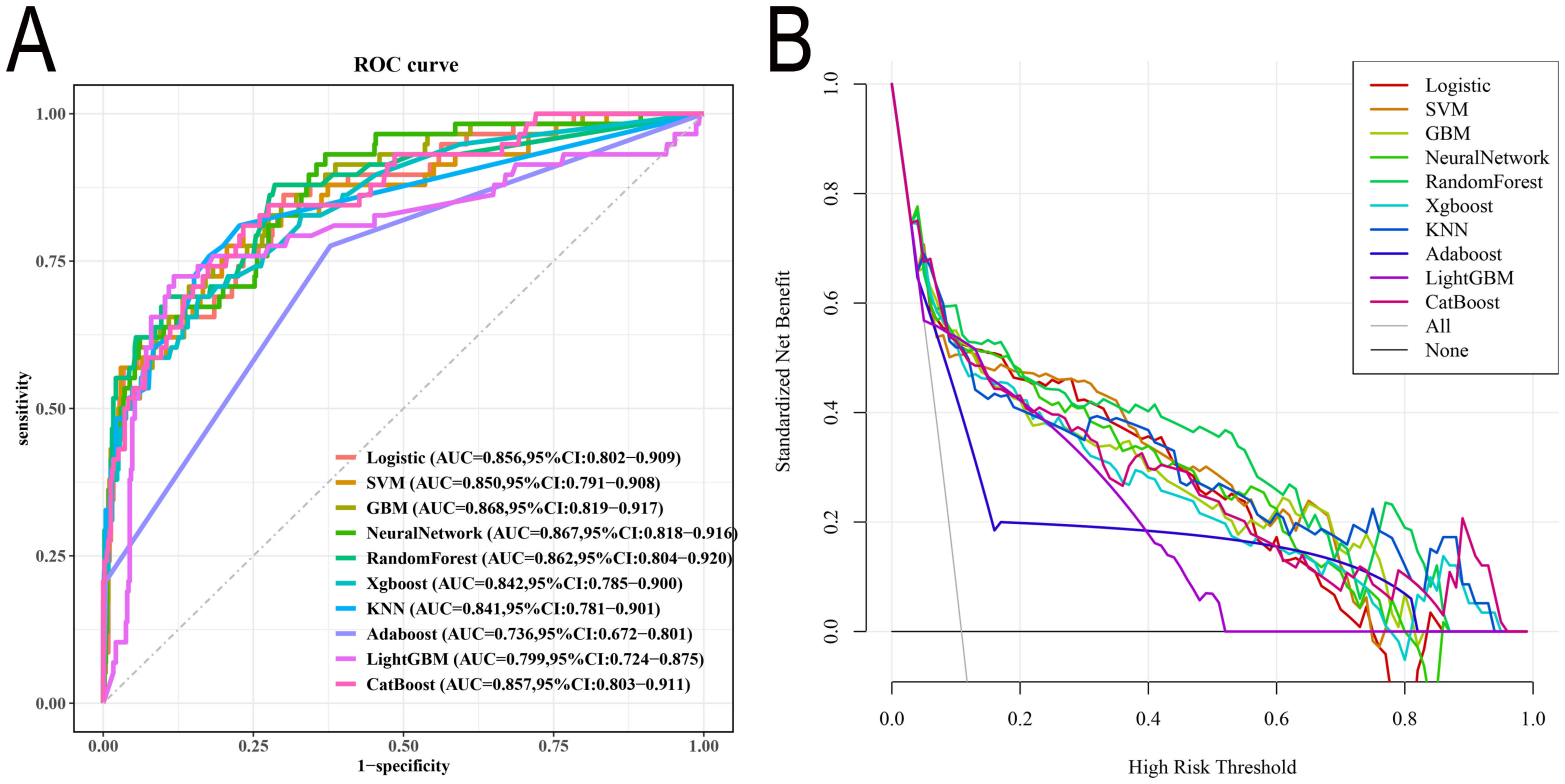
Machine learning model evaluation curves. **(A)** Comparison of ROC curves for machine learning models. Logistic, Logistic Regression; SVM, Support Vector Machine; GBM, Gradient Boosting Machine; Neural Network, Neural Network; Random Forest, Random Forest; XGBoost, Extreme Gradient Boosting; KNN, K-Nearest Neighbours; AdaBoost, Adaptive Boosting; LightGBM, Lightweight Gradient Boosting Machine; CatBoost, Categorical Boosting Algorithm. **(B)** Clinical Decision Curve Analysis (DCA). The x-axis represents the probability threshold for intervention selected by the clinician or decision maker, and the y-axis represents the net benefit of the decision strategy at that threshold. The higher the curve, the more effective the model.

The ROC analysis revealed that ensemble models generally performed well, with the GBM achieving the highest AUC (0.868, 95% CI: 0.819–0.917), closely followed by the Neural Network (AUC = 0.867). In comparison, the AdaBoost (AUC = 0.736) and LightGBM (AUC = 0.799) models yielded lower predictive accuracy in this context (**Figure 3A**). Decision curve analysis (DCA) further supported the clinical applicability of the GBM, demonstrating a relatively high net benefit across a range of threshold probabilities (**Figure 3B**). At an optimal probability threshold of 0.15, the GBM model achieved an accuracy of 0.905, a sensitivity of 0.919, a specificity of 0.904, a precision of 0.539, an F1-score of 0.679, and Brier score of 0.076 (**Table 3**). Calibration was assessed using Brier score and calibration curves (**Table 3**; **Supplementary Figure 3**). The GBM model showed acceptable calibration (Brier score = 0.076), and its calibration curve closely followed the diagonal.

**Table 3:** Comparison of machine learning model performance (test dataset).

Model	Threshold	Accuracy	Sensitivity	Specificity	Precision	F1	Brier score
Logistic	0.04	0.717	0.862	0.700	0.259	0.398	0.068
SVM	0.04	0.772	0.810	0.767	0.297	0.435	0.072
GBM	0.11	0.841	0.707	0.857	0.376	0.491	0.076
NeuralNetwork	0.03	0.663	0.931	0.630	0.235	0.375	0.066
RandomForest	0.05	0.732	0.879	0.714	0.273	0.416	0.067
Xgboost	0.50	0.824	0.690	0.840	0.345	0.460	0.247
KNN	0.01	0.777	0.810	0.773	0.303	0.441	0.067
Adaboost	0.17	0.639	0.776	0.622	0.200	0.318	0.082
LightGBM	0.01	0.865	0.724	0.882	0.429	0.538	0.085
CatBoost	0.52	0.772	0.810	0.767	0.297	0.435	0.258

### SHAP-based model interpretability analysis

**Figure 4:**
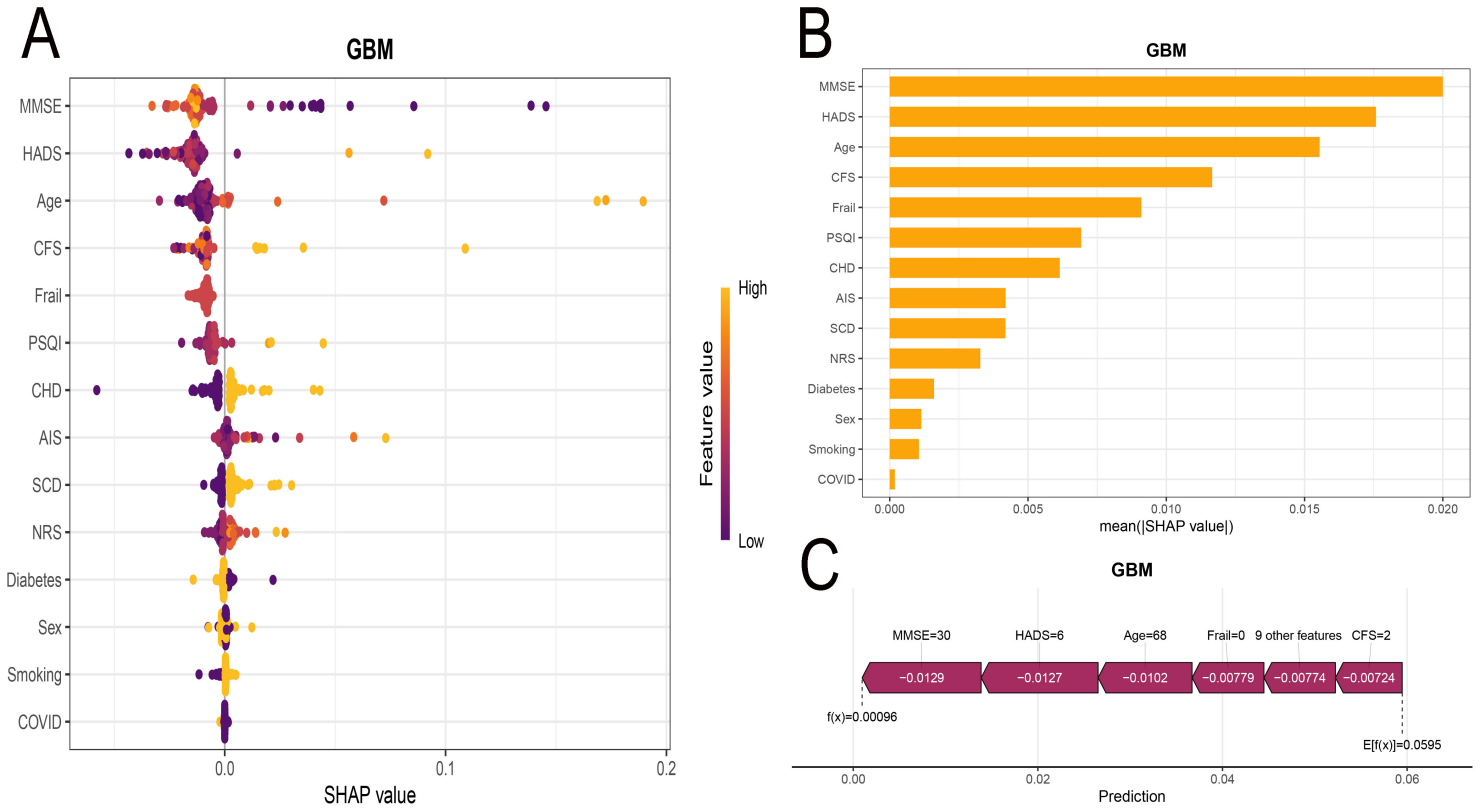
Demographic and clinical feature selection using the LASSO regression. **(A)** SHAP summary plot of the GBM model. The color represents the value of the variable. **(B)** SHAP importance for each feature of the GBM model. **(C)** SHAP force plot of the GBM model.

To elucidate the decision-making process of the GBM model, SHAP analysis was employed to quantify feature contributions. The summary plot and importance ranking identified MMSE score as the most influential predictor, followed by HADS score, age, and geriatric vulnerabilities including CFS, Frail index, and PSQI (**Figures 4A, B**).

SHAP summary plots revealed that lower MMSE scores and elevated HADS scores exhibited the strongest positive attribution to POD risk. Notably, the model captured non-linear trends, with risk increasing significantly as cognitive reserve diminishes and psychological distress intensifies. Furthermore, individual force plots demonstrate the specific direction and magnitude of feature contributions for representative cases. For instance, in a patient with an MMSE of 30 and HADS of 6, these factors exerted a protective effect (SHAP values < 0), collectively driving the predicted risk below the baseline expectation (**Figure 4C**).

### Nomogram development and logistic regression model-based application for POD

**Figure 5:**
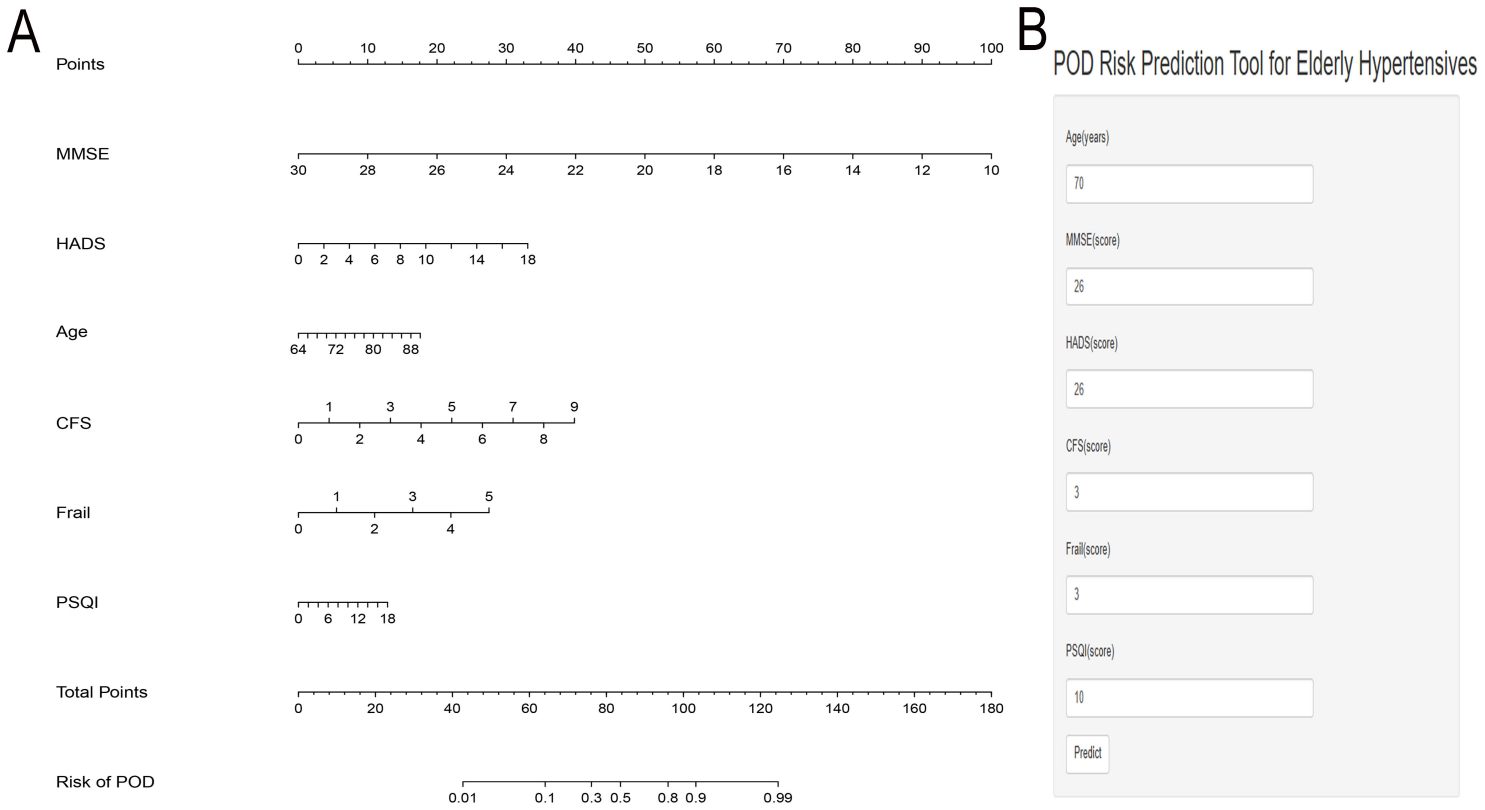
Nomogram development and logistic regression model-based application for POD. **(A)** To estimate the POD risk, the value for each clinical variable is aligned with its corresponding “Points” axis at the top to obtain an individual score. The scores for all six variables (MMSE, HADS, Age, CFS, Frail, PSQI) are summed to yield a total points value. This total is then projected downward to the “Risk of POD” axis at the bottom to read the corresponding predicted probability. **(B)** The patient’s clinical parameters are entered into an online tool developed using the underlying logistic regression model. The platform automatically processes the inputs and generates a real-time POD risk estimate.

The discriminative performance of our six-predictor (age, MMSE, HADS, CFS, Frail, PSQI scores) logistic regression model was satisfactory, yielding an AUC of 0.835 (95% CI: 0.776–0.895) (**Supplementary Figure 2**). For clinical application, we presented the model as a visual nomogram to facilitate individualized POD risk estimation (**Figure 5A**). Additionally, we created an openly accessible, interactive web platform (https://wangkun.shinyapps.io/make_web/) that implements this model for real-time risk calculation (**Figure 5B**).

### Survival analysis

**Figure 6:**
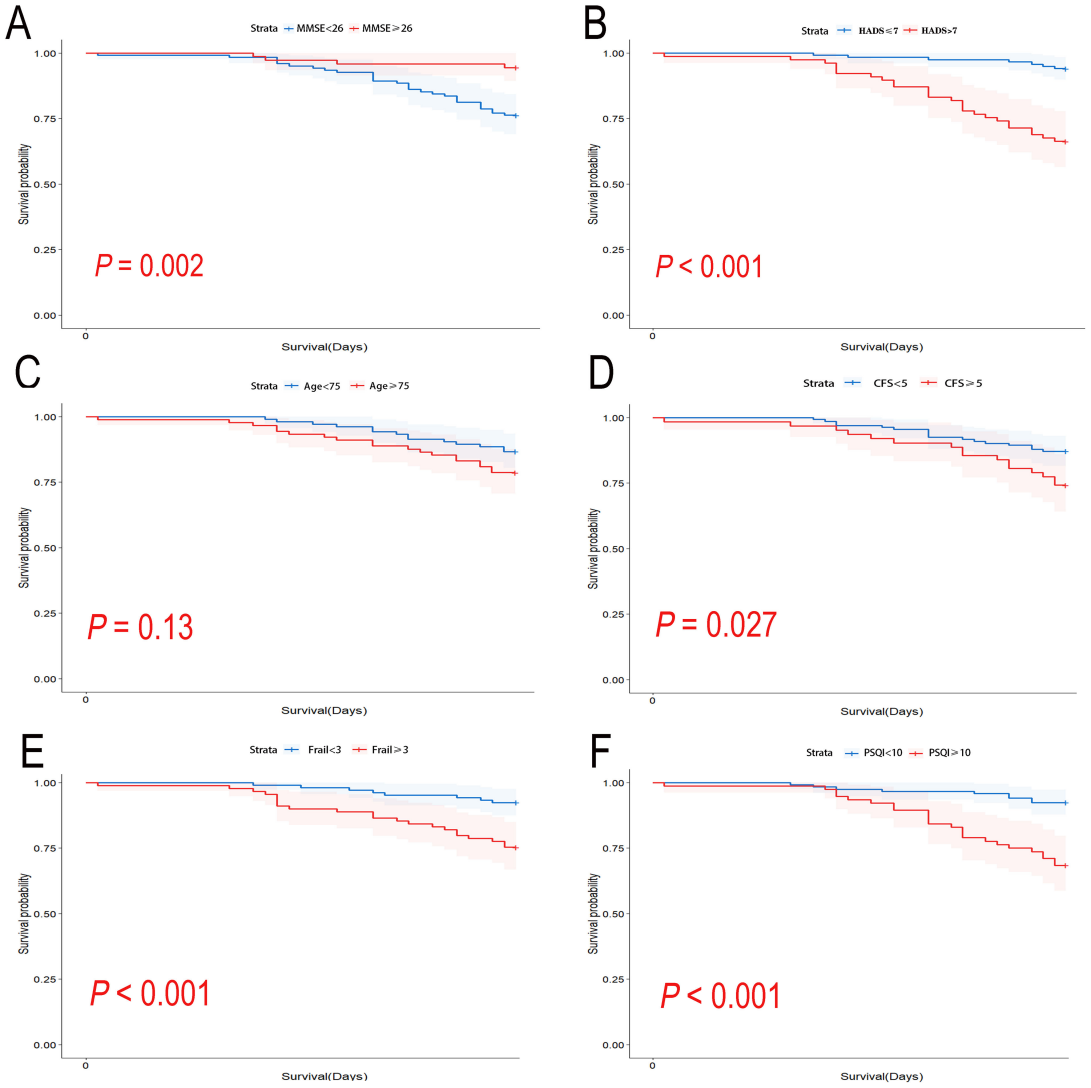
Kaplan-Meier survival curves comparing three-year survival probabilities in patients with POD. **(A)** Survival probability stratified by MMSE score. **(B)** Survival probability stratified by HADS score. **(C)** Survival probability stratified by age. **(D)** Survival probability stratified by CFS score. **(E)** Survival probability stratified by Frail Scale score. **(F)** Survival probability stratified by PSQI score. Red texts indicate P-values from Log-rank tests. Abbreviation: POD, postoperative delirium; MMSE, Mini-Mental State Examination; HADS, Hospital Anxiety and Depression Scale; CFS, Clinical Frailty Scale; PSQI, Pittsburgh Sleep Quality Index.

To identify determinants of long-term prognosis, we performed a survival analysis within the POD cohort. Initial Kaplan-Meier analysis showed that lower MMSE (*P* = 0.002), higher HADS (*P* < 0.001), higher CFS (*P* = 0.027), higher Frailty scores (*P* < 0.001), and higher PSQI scores (*P* < 0.001) were associated with increased three-year mortality (**Figure 6**).

Multivariable Cox regression, adjusted for demographics (sex, smoking), preoperative comorbidities (CHD, diabetes), and clinical status (NRS, COVID-19, AIS, and SCD), identified several independent risk factors. These included cognitive impairment (MMSE: HR = 0.250, 95% CI: 0.083–0.752, *P* = 0.014), psychological distress (HADS: HR = 16.014, 95% CI: 5.181–49.495, *P* < 0.001), frailty (CFS: HR = 2.296, 95% CI: 1.088–4.847, *P* = 0.029; Frail: HR = 2.799, 95% CI: 1.290–6.071, *P* = 0.009), and poor sleep quality (PSQI: HR = 4.847, 95% CI: 1.913–12.280, *P* < 0.001). In contrast, advanced age was not significantly associated with three-year mortality in either the Kaplan–Meier analysis (*P* = 0.13) or the multivariable Cox regression (HR = 2.202, 95% CI: 0.972–4.987, *P* = 0.059) (**Table 4**).

**Table 4:** Factors associated with three-year mortality in patients with POD.

Variables	Category	HR (95% CI)	*P* value
MMSE score	<26	0.250(0.083-0.752)	**0.014**
	≥26		
HADS score	≤7	16.014(5.181-49.495)	**<0.001**
	>7		
Age	<75 years	2.202(0.972-4.987)	0.059
	≥75 years		
CSF score	<5	2.296(1.088-4.847)	**0.029**
	≥5		
Frail score	<3	2.799(1.290-6.071)	**0.009**
	≥3		
PSQI score	<10	4.847(1.913-12.280)	**0.001**
	≥10		

HR, hazard ratio; CI, confidence interval; The bold values are all *p* ≤ 0.05, statistically significant parameters.

## Discussion

This study developed and validated machine learning models to predict POD in elderly hypertensive patients undergoing non-cardiac surgery. Among the ten models evaluated, the GBM demonstrated the best predictive performance, achieving an AUC of 0.868 (95% CI: 0.819–0.917) and superior DCA net benefit. The model incorporated 14 routinely accessible preoperative variables, providing a clinically practical tool for individualized preoperative risk stratification.

The strong performance of the GBM model can be attributed to its ability to capture complex, nonlinear interactions through sequential ensemble learning (19). While the Neural Network showed a comparable AUC, GBM achieved a superior F1-score and higher net benefit in DCA, highlighting its clinical utility. These results align with our previous findings; however, the current model enhances clinical feasibility by replacing invasive CSF biomarkers with routinely accessible scales (20). Compared to an external study using GBM for POD prediction (AUC = 0.80, n=797) (13), our study utilized a substantially larger cohort (n=1782) and achieved a higher test-set AUC of 0.868 (95% CI: 0.819–0.917). The EPV ratio of 13.9:1 further indicates a low risk of overfitting. This research represents one of the most comprehensive efforts to develop a non-invasive, multidimensional prediction tool specifically for elderly hypertensive patients. The rigorous validation via ROC and DCA reinforces the model’s discriminative capacity and potential to provide meaningful net clinical benefits across diverse risk thresholds.

SHAP analysis identified the MMSE score as the most influential predictor, followed by HADS, consistent with the stress–vulnerability model. Low MMSE reflects reduced neural reserve, impairing tolerance to surgical stress (21, 22). Elevated HADS may trigger HPA axis activation and neuroendocrine dysregulation, exacerbating susceptibility to POD (23–26). Furthermore, our multivariable Cox regression analysis—a significant extension requested to strengthen the evaluation of long-term prognosis—demonstrated that preoperative cognitive impairment (MMSE), psychological distress (HADS), frailty (CFS/Frail), and poor sleep quality (PSQI) are robust independent predictors of three-year mortality within the POD population. Notably, consistent with our Kaplan-Meier analysis, chronological age did not emerge as a significant independent risk factor in the multivariable model, suggesting that functional and psychological resilience may be more critical than biological age in determining the long-term survival of POD patients (27–29).

Specifically, the association between lower MMSE, higher HADS, and increased mortality suggests that compromised neural and psychological reserve not only predisposes patients to acute delirium but also hampers the physiological recovery essential for long-term survival (30, 31). Psychological distress, as indicated by elevated HADS, may trigger prolonged neuroendocrine-immune dysregulation, creating a state of chronic stress that exacerbates underlying pathologies (32). Furthermore, the independent prognostic value of poor sleep quality (PSQI) and frailty (CFS/Frailty) underscores the role of systemic biological vulnerability. Chronic sleep fragmentation, reflected by high PSQI scores, is known to hinder neural repair and exacerbate systemic inflammation through NF-κB activation, thereby diminishing long-term resilience (33, 34). Similarly, frailty likely reflects underlying mitochondrial dysfunction and reduced biological capacity to withstand the catabolic stress of surgery (35). Collectively, these parameters define a “high-risk phenotype” characterized by multi-system exhaustion, which warrants integrated preoperative assessment and targeted interventions, such as cognitive prehabilitation, psychological counseling, and multimodal frailty programs.

However, several limitations must be acknowledged. First, this single-center retrospective study lacked external validation. The internal validation may overestimate performance, and the model’s generalizability to other populations remains unknown. Future multicenter prospective studies are needed to verify the model’s performance across diverse settings. Second, we excluded patients with preoperative MMSE <23, this exclusion may introduce selection bias and potentially overestimate model performance by removing the highest-risk subgroup. We implemented this threshold to ensure participants possessed the cognitive capacity to reliably complete subjective scales (e.g., HADS, PSQI); nevertheless, our findings may not be directly generalizable to patients with moderate-to-severe baseline dementia. Third, the current model relies on preoperative characteristics and does not yet incorporate dynamic intraoperative factors, such as hemodynamic variability or anesthetic depth, which could further refine risk stratification in future iterations.

## Conclusion

We developed a GBM-based machine learning model (AUC = 0.868) that accurately predicts POD in elderly hypertensive patients. MMSE, HADS, frailty, and PSQI were identified as key independent predictors for both POD and three-year mortality. These functional and psychological vulnerabilities—forming a “high-risk phenotype”—hold greater prognostic value for long-term survival than chronological age. Our results emphasize the necessity of multidimensional preoperative assessments for personalized risk management in this population.

## Data Availability

The raw data supporting the conclusions of this article will be made available by the authors, without undue reservation.

## References

[1] Ormseth CH LaHue SC Oldham MA Josephson SA Whitaker E Douglas VC . Predisposing and precipitating factors associated with delirium: a systematic review. JAMA Netw Open. (2023) 6:e2249950. doi: 10.1001/jamanetworkopen.2022.49950. PMID: 36607634 PMC9856673

[2] Sadeghirad B Dodsworth BT Schmutz Gelsomino N Goettel N Spence J Buchan TA . Perioperative factors associated with postoperative delirium in patients undergoing noncardiac surgery: an individual patient data meta-analysis. JAMA Netw Open. (2023) 6:e2337239. doi: 10.1001/jamanetworkopen.2023.37239. PMID: 37819663 PMC10568362

[3] Jaqua EE Nguyen VTN Chin E . Delirium in older persons: prevention, evaluation, and management. Am Family Physician. (2023) 108:278–87. 37725462

[4] Wang K Zhang A Kong W Wang Y Liang Y Lin Y . Association of cardiometabolic multimorbidity with postoperative delirium and three-year mortality in patients undergoing knee/hip arthroplasty: a prospective cohort study. Int J Surg (London England). (2025) 111:3821–30. doi: 10.1097/js9.0000000000002379. PMID: 40434729 PMC12165473

[5] Fong TG Tulebaev SR Inouye SK . Delirium in elderly adults: diagnosis, prevention and treatment. Nat Rev Neurol. (2009) 5:210–20. doi: 10.1038/nrneurol.2009.24. PMID: 19347026 PMC3065676

[6] Tang X Yv H Wang F Wang J Liu S Wu X . The relationship between suboptimal social networks and postoperative delirium: the PNDABLE study. Front Aging Neurosci. (2022) 14:851368. doi: 10.3389/fnagi.2022.851368. PMID: 35769605 PMC9235411

[7] Wang J Wang L Tang X Wang F Liu S Wu X . The relationship between cardiovascular disease risk score and postoperative delirium: the PNDABLE study. Front Aging Neurosci. (2022) 14:851372. doi: 10.3389/fnagi.2022.851372. PMID: 35800979 PMC9252852

[8] Inouye SK Westendorp RG Saczynski JS . Delirium in elderly people. Lancet (London England). (2014) 383:911–22. doi: 10.1016/s0140-6736(13)60688-1. PMID: 23992774 PMC4120864

[9] Clare L Wu YT Teale JC MacLeod C Matthews F Brayne C . Potentially modifiable lifestyle factors, cognitive reserve, and cognitive function in later life: a cross-sectional study. PloS Med. (2017) 14:e1002259. doi: 10.1371/journal.pmed.1002259. PMID: 28323829 PMC5360216

[10] Tonna JE Dalton A Presson AP Zhang C Colantuoni E Lander K . The effect of a quality improvement intervention on sleep and delirium in critically ill patients in a surgical ICU. Chest. (2021) 160:899–908. doi: 10.1016/j.chest.2021.03.030. PMID: 33773988 PMC8448998

[11] Wu Y Shi Z Wang M Zhu Y Li C Li G . Different MMSE score is associated with postoperative delirium in young-old and old-old adults. PloS One. (2015) 10:e0139879. doi: 10.1371/journal.pone.0139879. PMID: 26460750 PMC4603675

[12] Price CC Garvan C Hizel LP Lopez MG Billings F . Delayed recall and working memory MMSE domains predict delirium following cardiac surgery. J Alzheimer's Dis JAD. (2017) 59:1027–35. doi: 10.3233/jad-170380. PMID: 28697572 PMC5544543

[13] Song Y Zhang D Wang Q Liu Y Chen K Sun J . Prediction models for postoperative delirium in elderly patients with machine-learning algorithms and SHapley additive explanations. Transl Psychiatry. (2024) 14:57. doi: 10.1038/s41398-024-02762-w. PMID: 38267405 PMC10808214

[14] Xue B Li D Lu C King CR Wildes T Avidan MS . Use of machine learning to develop and evaluate models using preoperative and intraoperative data to identify risks of postoperative complications. JAMA Netw Open. (2021) 4:e212240. doi: 10.1001/jamanetworkopen.2021.2240. PMID: 33783520 PMC8010590

[15] Santisteban MM Iadecola C Carnevale D . Hypertension, neurovascular dysfunction, and cognitive impairment. Hypertension (Dallas Tex 1979). (2023) 80:22–34. doi: 10.1161/hypertensionaha.122.18085. PMID: 36129176 PMC9742151

[16] Hainsworth AH Markus HS Schneider JA . Cerebral small vessel disease, hypertension, and vascular contributions to cognitive impairment and dementia. Hypertension (Dallas Tex 1979). (2024) 81:75–86. doi: 10.1161/hypertensionaha.123.19943. PMID: 38044814 PMC10734789

[17] Santisteban MM Ahn SJ Lane D Faraco G Garcia-Bonilla L Racchumi G . Endothelium-macrophage crosstalk mediates blood-brain barrier dysfunction in hypertension. Hypertension (Dallas Tex 1979). (2020) 76:795–807. doi: 10.1161/hypertensionaha.120.15581. PMID: 32654560 PMC7429290

[18] Oliveira FR Oliveira VH Oliveira ÍM Lima JW Calderaro D Gualandro DM . Hypertension, mitral valve disease, atrial fibrillation and low education level predict delirium and worst outcome after cardiac surgery in older adults. BMC Anesthesiology. (2018) 18:15. doi: 10.1186/s12871-018-0481-0. PMID: 29390969 PMC5796436

[19] Neto PCS Rodrigues AL Stahlschmidt A Helal L Stefani LC . Developing and validating a machine learning ensemble model to predict postoperative delirium in a cohort of high-risk surgical patients: a secondary cohort analysis. Eur J Anaesthesiology. (2023) 40:356–64. doi: 10.1097/eja.0000000000001811. PMID: 36860180

[20] Liang Y Mu C Kong W Wang K Hua S Wang Y . Tau protein mediates the association between frailty and postoperative delirium: a machine learning model incorporating cerebrospinal fluid biomarkers. Front Neurol. (2025) 16:1608264. doi: 10.3389/fneur.2025.1608264. PMID: 41041678 PMC12483881

[21] Wang B Mu C Tang X Wang F Zhang G Wang J . The relationship between mild cognitive impairment and postoperative delirium undergoing total knee arthroplasty: the PNDABLE study. Front Aging Neurosci. (2022) 14:959510. doi: 10.3389/fnagi.2022.959510. PMID: 36247988 PMC9559362

[22] Amrapala A Sabé M Solmi M Maes M . Neuropsychiatric disturbances in mild cognitive impairment: a scientometric analysis. Ageing Res Rev. (2023) 92:102129. doi: 10.1016/j.arr.2023.102129. PMID: 37981054

[23] Yang KL Detroyer E Van Grootven B Tuand K Zhao DN Rex S . Association between preoperative anxiety and postoperative delirium in older patients: a systematic review and meta-analysis. BMC Geriatrics. (2023) 23:198. doi: 10.1186/s12877-023-03923-0. PMID: 36997928 PMC10064748

[24] Chen A An E Yan E Saripella A Khullar A Misati G . Prevalence of preoperative depression and adverse outcomes in older patients undergoing elective surgery: a systematic review and meta-analysis. J Clin Anesth. (2024) 97:111532. doi: 10.1016/j.jclinane.2024.111532. PMID: 38936304

[25] Luo T Deng Z Ren Q Mu F Zhang Y Wang H . Effects of esketamine on postoperative negative emotions and early cognitive disorders in patients undergoing non-cardiac thoracic surgery: a randomized controlled trial. J Clin Anesth. (2024) 95:111447. doi: 10.1016/j.jclinane.2024.111447. PMID: 38522144

[26] Zheng F Zhao Y Ren X Lan X Peng W . Prevalence and associated factors of cognitive frailty in older patients with lung cancer undergoing chemotherapy: a cross-sectional study. Supportive Care Cancer Off J Multinational Assoc Supportive Care Cancer. (2025) 33:762. doi: 10.1007/s00520-025-09821-y. PMID: 40775088

[27] Pinho C Cruz S Santos A Abelha FJ . Postoperative delirium: age and low functional reserve as independent risk factors. J Clin Anesth. (2016) 33:507–13. doi: 10.1016/j.jclinane.2015.09.002. PMID: 26604015

[28] Persico I Cesari M Morandi A Haas J Mazzola P Zambon A . Frailty and delirium in older adults: a systematic review and meta-analysis of the literature. J Am Geriatrics Soc. (2018) 66:2022–30. doi: 10.1111/jgs.15503. PMID: 30238970

[29] Huang Y Chen G Ma N Wang L Wang Y Jia Z . Associations of frailty status and sleep quality with incident delirium: a prospective study in the UK Biobank. CNS Neurosci Ther. (2025) 31:e70266. doi: 10.1111/cns.70266. PMID: 39992050 PMC11848733

[30] Lobo E De la Cámara C Gracia-García P Saz P López-Antón R Lobo A . Cognitive trajectories in older adults and associated mortality and predictors. Soc Psychiatry Psychiatr Epidemiol. (2025) 60:2887–95. doi: 10.1007/s00127-025-02862-y. PMID: 40047850 PMC12594731

[31] van Dijk MR Utens EM Dulfer K Al-Qezweny MN van Geuns RJ Daemen J . Depression and anxiety symptoms as predictors of mortality in PCI patients at 10 years of follow-up. Eur J Prev Cardiol. (2016) 23:552–8. doi: 10.1177/2047487315571889. PMID: 25665581

[32] Fan J Yu C Guo Y Bian Z Sun Z Yang L . Frailty index and all-cause and cause-specific mortality in Chinese adults: a prospective cohort study. Lancet Public Health. (2020) 5:e650-e660. doi: 10.1016/s2468-2667(20)30113-4. PMID: 33271078 PMC7708389

[33] Shi SM Olivieri-Mui B McCarthy EP Kim DH . Changes in a frailty index and association with mortality. J Am Geriatrics Soc. (2021) 69:1057–62. doi: 10.1111/jgs.17002. PMID: 33377190 PMC8071066

[34] Watanabe D Yoshida T Watanabe Y Yamada Y Miyachi M Kimura M . Combined use of sleep quality and duration is more closely associated with mortality risk among older adults: a population-based Kyoto-Kameoka prospective cohort study. J Epidemiol. (2023) 33:591–9. doi: 10.2188/jea.je20220215. PMID: 36155361 PMC10635816

[35] Veronese N Custodero C Demurtas J Smith L Barbagallo M Maggi S . Comprehensive geriatric assessment in older people: an umbrella review of health outcomes. Age Ageing. (2022) 51. doi: 10.1093/ageing/afac104. PMID: 35524746

