# Supplementary figures and images for: Prediction model for postoperative delirium risk in elderly hypertensive patients: machine learning-based development and validation
Source: Front Psychiatry. 2026 Jun 3;17:1851947. doi: 10.3389/fpsyt.2026.1851947 (PMC13272456; doi:10.3389/fpsyt.2026.1851947)

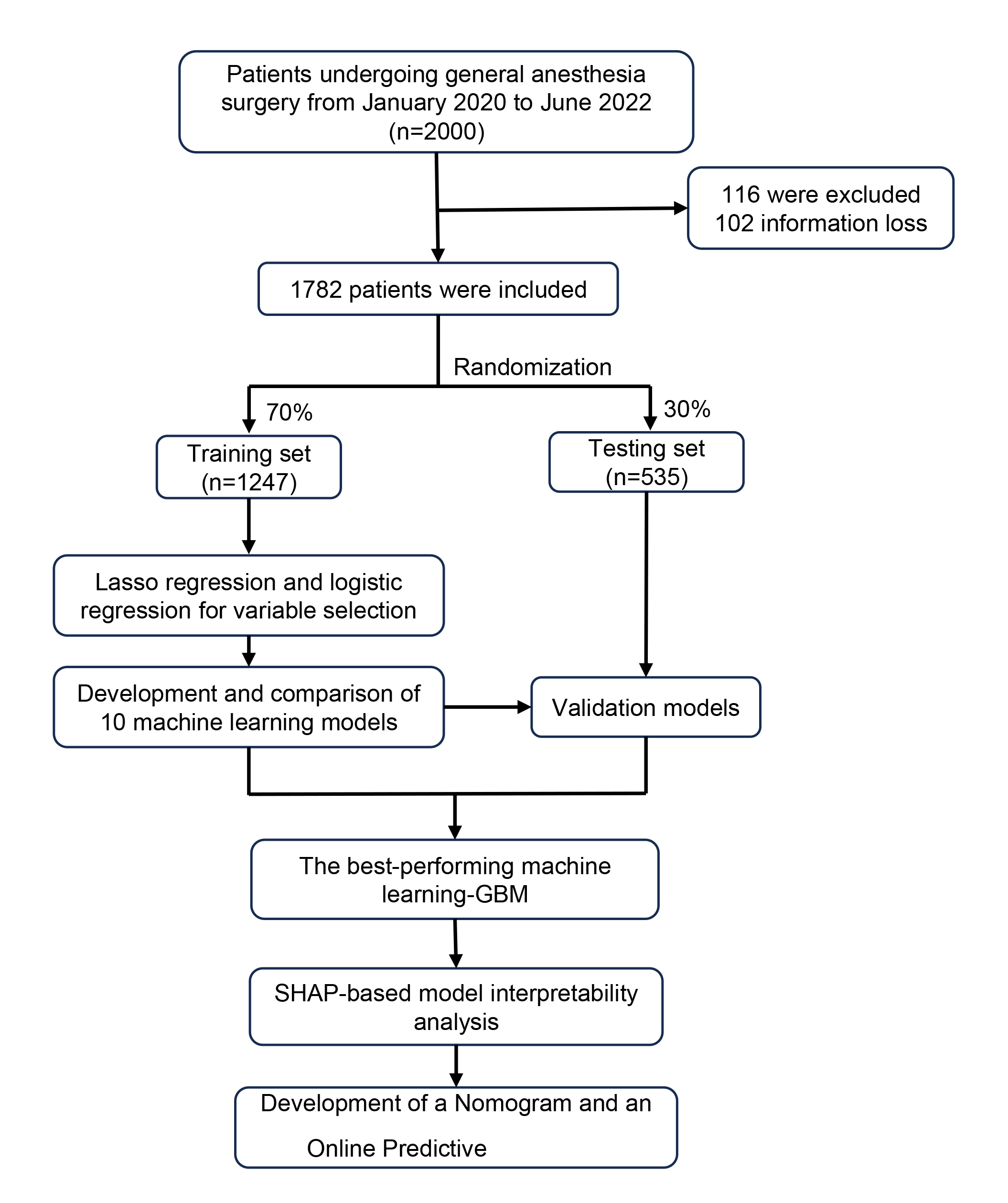

Supplement: Supplementary file 1 [file Image1.tif]

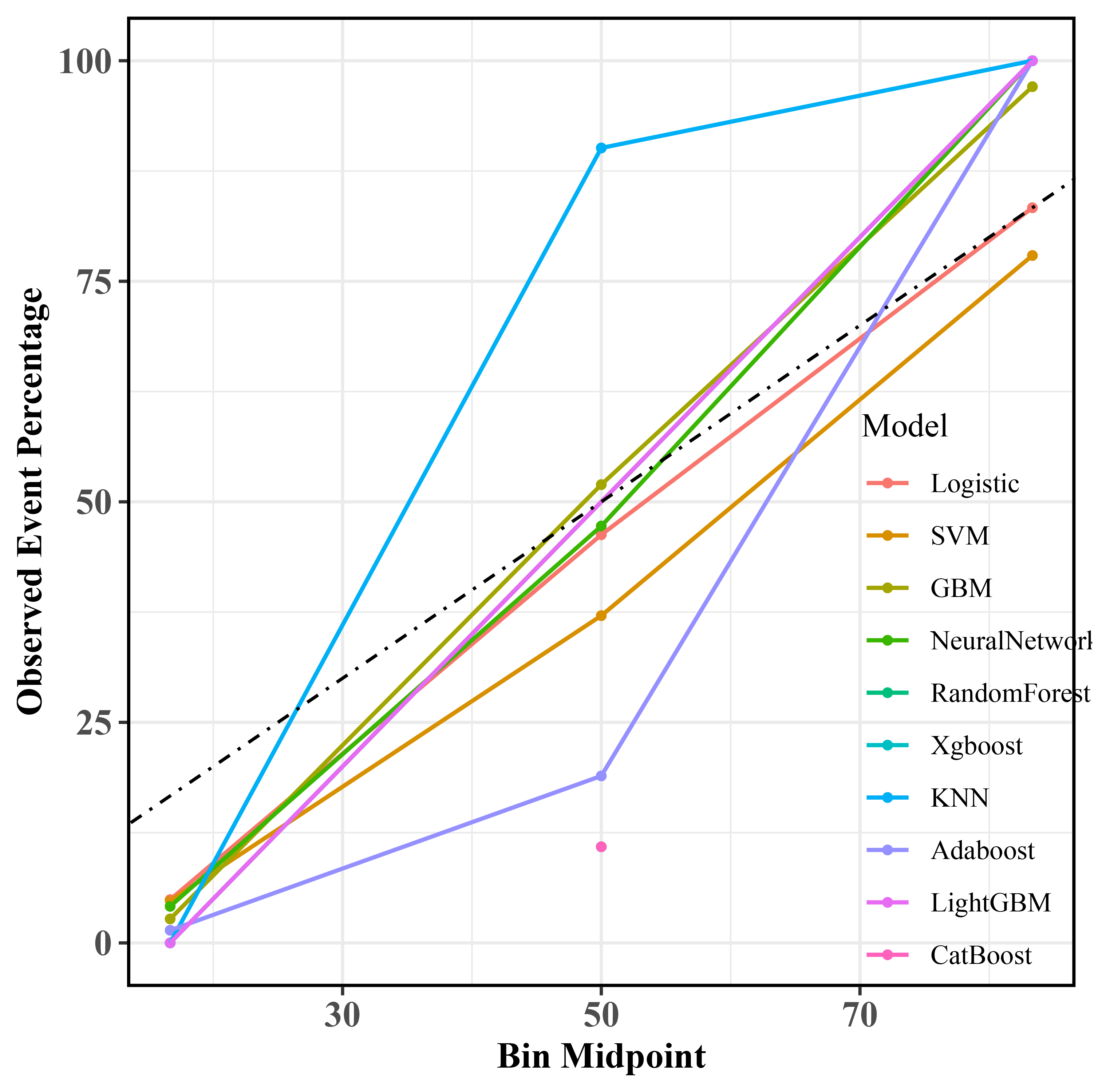

Supplement: Supplementary file 3 [file Image3.tif]
